# An Automatic Localization Algorithm for Ultrasound Breast Tumors Based on Human Visual Mechanism

**DOI:** 10.3390/s17051101

**Published:** 2017-05-11

**Authors:** Yuting Xie, Ke Chen, Jiangli Lin

**Affiliations:** Department of Biomedical Engineering, Sichuan University, Chengdu 610065, China; 2014223010060@stu.scu.edu.cn (Y.X.); chenke@scu.edu.cn (K.C.)

**Keywords:** automatic localization, human visual mechanisms, superpixel contrast feature, ultrasound breast tumor

## Abstract

Human visual mechanisms (HVMs) can quickly localize the most salient object in natural images, but it is ineffective at localizing tumors in ultrasound breast images. In this paper, we research the characteristics of tumors, develop a classic HVM and propose a novel auto-localization method. Comparing to surrounding areas, tumors have higher global and local contrast. In this method, intensity, blackness ratio and superpixel contrast features are combined to compute a saliency map, in which a Winner Take All algorithm is used to localize the most salient region, which is represented by a circle. The results show that the proposed method can successfully avoid the interference caused by background areas of low echo and high intensity. The method has been tested on 400 ultrasound breast images, among which 376 images succeed in localization. This means this method has a high accuracy of 94.00%, indicating its good performance in real-life applications.

## 1. Introduction

Breast cancer is one of the most common malignant tumors women face. It has the highest incidence and mortality of all diseases affecting women and even holds a rising trend. The early detection of suspicious lesions is very important for effective treatment of breast cancer. Ultrasound, mammography and magnetic resonance imaging (MRI) are general methods for the clinical detection of lesions, among which ultrasound (US) has been widely used due to its non-invasion, non-ionization radiation and non-injury [1,2]. However, the shortcomings of US breast images, such as low contrast, serious speckles, and low spatial resolution, make it difficult for doctors to read and analyze these suspicious lesions. Furthermore, with an increasing number of patients, doctors feel heavily burdened, resulting in a higher rate of misdiagnosis.

In recent years, to improve the usability of US computer-aided diagnosis (CAD) has been preferred to achieve more reliable and accurate diagnostic conclusions and to reduce the unnecessary MRI and biopsies. In addition, CAD techniques used to investigate suspicious lesions in US breast images can also help reduce the workload of doctors [3,4,5,6]. Generally, a CAD system mainly contains three steps: image segmentation, feature extraction and object classification. Image segmentation is an essential step. In recent decades, many segmentation techniques have been proposed [7], such as neural network (NN)-based methods [8,9], deep learning, active contour models [6], and region-based methods [10,11]. For these segmentation algorithms, due to the interference caused by shadowing artifacts or similar tumor-like structures for the lesion, the automatic localization of tumors in US breast images is a difficult and critical link [8,9,10,11]. In fact, generally, lesions are manually marked as an alternative method, but artificial intervention makes it impossible to realize fully automatic segmentation. 

To date, several methods have been proposed for the automatic localization of breast tumors in US images. Upon review of these methods, they can be generally classified into two types: point-based methods [10,11,12] and region-based methods [13,14,15].

Madabhushi et al. [11] proposed an automatic localization method using seeds point. This is a type of point-based method. In Madabhushi’s method, the reference point is generated based on empirical rules, with which suspicious lesions are more likely to be positioned at or near the center of an image. As their seed point selection method combines the probability distribution functions (pdfs) for intensity, texture and the location of the potential seed points, it is very likely to result in an incorrect location, where the true tumor is not located at the center of the image, or pixels with similar pdfs near the center of the image. Unfortunately, these two issues persist in US breast images. Therefore, the reference point assumption is ineffective and influenced by the operation habits of physicians. Besides, this point-based method is sensitive to speckle noise.

Other researchers have proposed region-based detection methods. Yap et al. [14] considered two factors for selection object region: the size and the distance of each region to the reference point. In this method, histogram equalization is firstly used to pre-process the images, which is followed by hybrid filtering and multifractal analysis stages. Then, the region of interest (ROI) is identified by a single-valued threshold segmentation method and a rule-based approach. Compared to point-based methods, this method has better performance in case of noise immunity. However, if the tumors are particularly small or the background areas are complex with serious artifacts, this method loses efficacy.

Liu [15] also presented a region-based detection method based on a support vector machine (SVM) classifier. He divided the images into 16 × 16 blocks and calculated the Gray Level Co-occurrence Matrix (GLCM) of each region as the training data of his SVM classifier. However this localization method needs many post-processing operations to select the ROI, including removing linear connected areas, filling holes and experience-based region selection. This method cannot separate tumor areas from normal tissues if only depending on the local texture feature. In addition, this method is unable to localize tumors in small-sized tumors and large-sized tumors shown in Section 4. 

In this paper, we propose a new localization method combining the point-based and region-based methods. This is based on the Itti HVM model [16]. Itti HVM model is a classic visual attention model, which has been widely applied to localize the most salient region in natural images by combining color, intensity, and orientation features. According to the characteristics of tumors in US images, we used the intensity and blackness ratio feature to compute the saliency map. In addition, in order to avoid the drawbacks of point-based methods, we add the superpixel feature so that these three features greatly improve the performance of this model, especially for the large and relatively small tumors. Finally, the automatically localized region is presented for the initial contour of the Chan-Vese (CV) level set. We employed the automatic segmentation of a breast tumor to demonstrate the practical significance of the proposed method.

## 2. Model Architecture

The human eye, working as a biological photoelectric sensor, can quickly obtain visual information. This information can stimulate the visual light-sensitive cells, producing electrical pulses, and guiding the eye movement. Different visual information causes different stimulation on the visual light-sensitive cells, with the most significant information gaining the attention of the human eyes. HVM has been successfully applied in the field of image processing [16,17,18,19,20], which can quickly localize the ROI, and automatically discard the unimportant parts. Thus, this saves a lot of image post-processing time.

**Figure 1:**
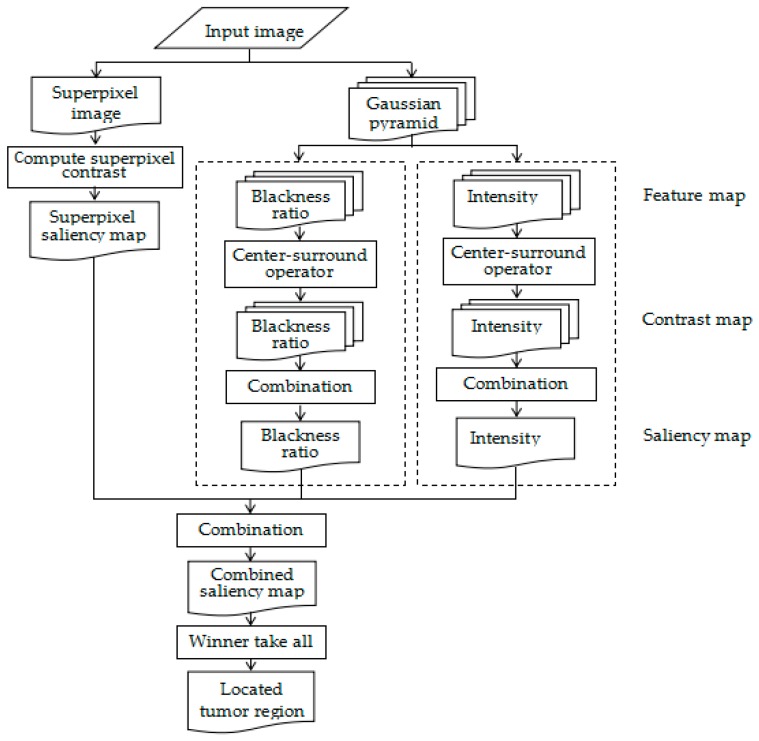
Proposed methodology.

In HVM, the movement of human eyes is mainly determined by visual features. Therefore, in HVM models, feature extraction is the most important part. For natural images, there are some features successfully applied in HVM models, such as color, texture and orientation. However, many features are usually invalid for US images. For instance, tumors are usually dark and directional features are too weak to distinguish the tumors from other dark areas. In our model, we used intensity, blackness ratio and superpixel contrast features to achieve tumor detection. This localization method can be divided into four steps: (a) getting feature maps and saliency maps of blackness ratio and intensity; (b) computing the superpixel saliency map of input image; (c) combining all saliency maps; and (d) locating the most salient region. The framework of the automated object localization method is shown in Figure 1.

### 2.1. Gaussian Pyramid Image

**Figure 2:**
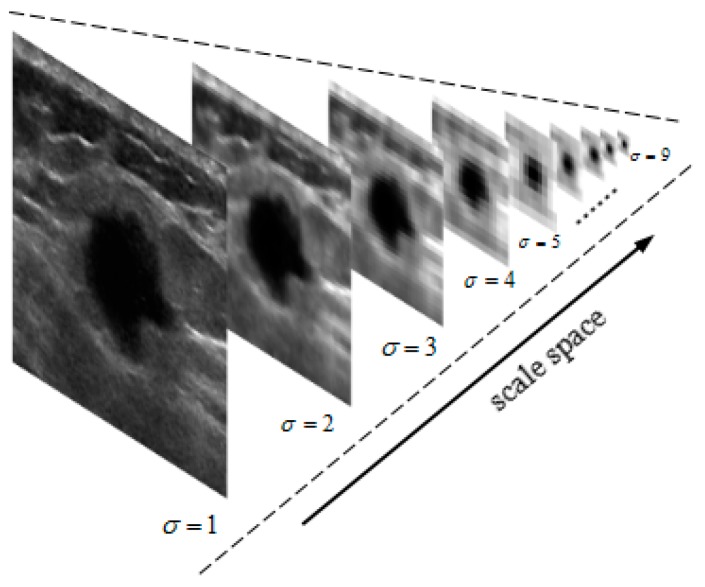
Gaussian pyramid of an input image.

In this algorithm, to avoid information loss through sub-sampling process, the input image is first smoothed by convolution with a linearly separable Gaussian filter and then sub-sampled by a factor of two [19]. In our model, we use a 6 × 6 linearly separable Gaussian kernel filter of [1,5,10,10,5,1,1]/32. The next levels σ=[1,2,...,9] of the pyramid are obtained by repeating the sub-sampling and decimation processes. σ=1 represents the input image. The resolution of layer σ is the $1/2^{\sigma }$ times of the original image, as this representation can effectively reduce time complexity of the algorithm. The Gaussian pyramid of input image is shown in Figure 2, in which images in higher levels have lower resolution, while the information in the object area is well retained, overall improving the efficiency of the procedure.

### 2.2. Saliency Map

#### 2.2.1. Intensity and Blackness Ratio Features

In US breast images, the intensity of tumors is lower than that of its surrounding areas, so tumors have great contrast to backgrounds. We obtained the intensity feature MI by computing the average of three color components:

(1) MI=(r+g+b)/3

US images are intrinsically gray-scale images, so the color components r, g and b are equal. For the record, the US breast images we obtained are stored in 24-bite bitmap (BMP) format, a standard image file format in Window operating system. In these images, the color components r, g and b have weak differences in gray values.

In addition, we defined the blackness ratio feature, in order to enlarge the weight of lower intensity, and weaken the influence of high-intensity areas. The blackness ratio map MBR is explained as

(2) $$MBR=\frac{1-\min\left(r,g,b\right)}{\left(r+g+b\right)/3+\varepsilon }$$

To avoid the divisor to be zero, ε is added in Equation (2), where ε is set to 0.001.

#### 2.2.2. Pyramid Feature Map

This involved extracting the intensity and blackness ratio features of each Gaussian pyramid image and repeating for pyramid levels σ=[1,2,...,9] to get the intensity pyramid feature map MI(σ) and blackness ratio pyramid feature map MBR(σ).

#### 2.2.3. Generating Saliency Map

**Figure 3:**
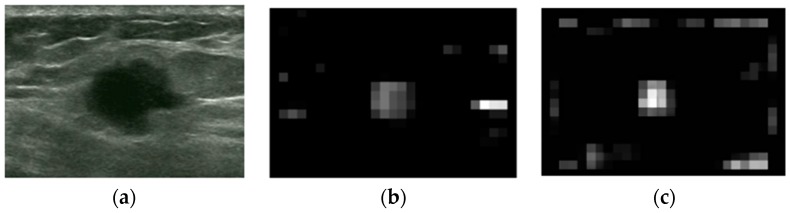
Saliency maps of intensity and blackness ratio features: (**a**) Original image. (**b**) Intensity saliency map. (**c**) Blackness ratio saliency map.

Center-surround receptive fields are simulated by across-scale subtraction between two maps at the center (c) and the surround (s) levels in these pyramids, yielding intensity and blackness ratio contrast maps [16,21,22].

(3) $$\begin{array}{l} CI\left(c,s\right)=N\left\{\left|MI\left(c\right)\Theta MI\left(s\right)\right|\right\}\\ CBR\left(c,s\right)=N\left\{\left|MBR\left(c\right)\Theta MBR\left(s\right)\right|\right\} \end{array}$$

where N{.} is an iterative, nonlinear normalization operator, simulating local competition between neighboring salient localization. Between one and five iterations are used for the simulations in this paper. The contrast maps are summed over by across-scale addition ⊕ to the σ=5 level. Following this, the sums are normalized again to generate a saliency map, as shown in Figure 3.

(4) $$\begin{array}{l} SI=N\left(\overset{5}{\underset{c=3}{⊕}}\overset{c+4}{\underset{s=c+3}{⊕}}CI\right)\\ SBR=N\left(\overset{5}{\underset{c=3}{⊕}}\overset{c+4}{\underset{s=c+3}{⊕}}CBR\right) \end{array}$$

In Figure 3b, a high-intensity area has higher contrast than tumor area, but the tumor area is more salient than other high-intensity regions whose values are near to zero. In the blackness ratio saliency map shown in Figure 3c, the tumor area has the highest saliency compared to any other regions.

When the local contrast of tumors is smaller than that of some background areas or speckle noise, the blackness ratio and intensity features are unable to localize the tumors. In order to avoid the interference of speckle noise and some background areas, we propose a novel global saliency feature, superpixel saliency, to enhance the saliency of tumors.

### 2.3. Superpixel Saliency

#### 2.3.1. Superpixel

A superpixel is a region that consists of a series of adjacent features with similar color and brightness. Most of these small areas retain the effective information for further image processing and generally preserve the boundaries of the object. We used simple linear iterative clustering (SLIC) to generate a superpixel image [23], in which the gray values and Euclidean distance are respectively used to compute the color similarity and spatial proximity of two pixels. Compared to existing superpixel methods, SLIC has been shown to outperform in respect to algorithm speed, memory efficiency and boundary adherence. Furthermore, the SLIC algorithm has excellent performance. In the SLIC algorithm, parameter K of K-means and compactness m are of importance to the segmentation performance. We discussed the segmentation performance of variant K and m.

**Figure 4:**
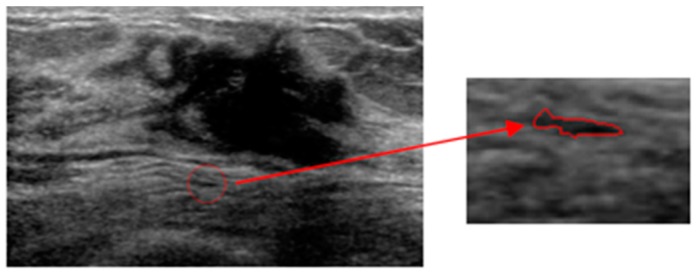
Input image of SLIC network.

**Figure 5:**
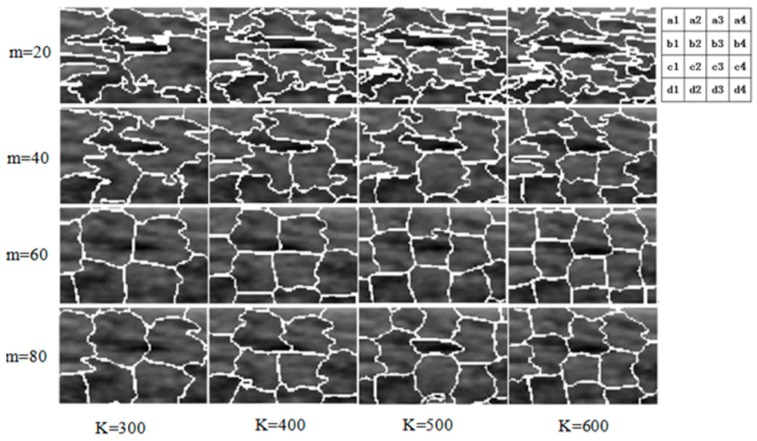
Performance comparison of SLIC with variant K and m of superpixels. These pictures are the superpixel images of a local region in a US breast image.

Combined with the reference segmentation results in Figure 4, we can see from the four lines in Figure 5 that compactness comes at the expense of boundary adherence. In other words, a higher compactness indicates more missed edges. Furthermore, as shown in Figure 5b1–b4 and c1–c4, the boundary adherence increases with K and then decreases rapidly when K > 400, resulting in poor segmentation performance. In our method, we choose the parameters K = 400 and m = 40, with which the SLIC algorithm can segment the locally pointed region well as shown in Figure 5b3 and the reference segmented region in Figure 4. 

**Figure 6:**
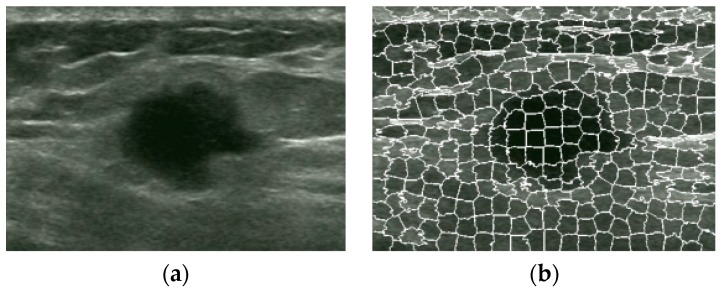
Superpixel image obtained by SLIC: (**a**) Original image. (**b**) Superpixel image.

The superpixel segmentation of the original image is shown in Figure 6b.

#### 2.3.2. Superpixel Saliency Map

The superpixel saliency map is computed as

(5) $$SSP\left(rk\right)=W\left(rk\right)\sum_{i=1,i\neq k}^{K}\left\{\frac{ni}{N}e^{-\frac{d^{2}\left(rk,ri\right)}{2\delta^{2}}}D\left(rk,ri\right)\right\},k=1,2,...,K$$

(6) $$W\left(rk\right)=\frac{\max\left(g\right)-gk}{\left(\max\left(g\right)-\min\left(g\right)\right)},g=\left\{g1,g2,...,gK\right\}$$

(7) $$d\left(rk,ri\right)=\sqrt{{\left(xk-xi\right)}^{2}+{\left(yk-yi\right)}^{2}}$$

(8) D(rk,ri)=gk−gi

In Equation (5), K is the number of superpixels; N and ni are respectively the total number of pixels in input image and in superpixel ri; δ is used to control the influence of surrounding superpixels on the superpixel being computed. In Equations (6) and (8), g is the set of average gray values gk of all superpixels. (xk,yk) and (xi,yi) in Equation (6) are the centroids of rk and ri, with d(rk,ri) then normalized to a range of [0,1]. 

In our method, three factors are considered: (a) Compared with normal tissues, a tumor has lower echo that makes it more salient than other areas, which is achieved by the weight W(rk). (b) Closer superpixels have a greater influence on the contrast of the current superpixel, as illustrated by the parameter $e^{-\frac{d^{2}\left(rk,ri\right)}{2\delta^{2}}}$ in Equation (5), in which δ controls the influence of surrounding superpixels ri on the current superpixel rk with respect to Euclidean distance, while δ is set to 0.05 to reduce the contribution of far superpixels. (c) Larger areas denote a greater effect on other areas, as illustrated with parameter $\frac{ni}{N}$.

**Figure 7:**
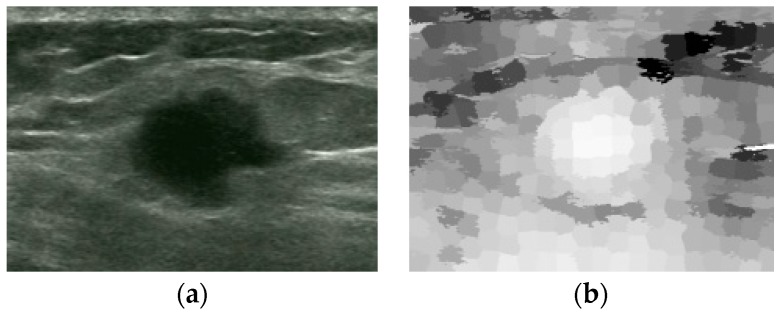
Original image and superpixel saliency map: (**a**) Original image. (**b**) Superpixel saliency map.

In superpixel saliency map in Figure 7b, the superpixels in tumor area all have relatively high contrast. Other areas, such as the marked high-intensity area, are less salient because of their small areas.

### 2.4. Combined Saliency Map

(9) $$S=\frac{1}{3}\sum_{l\in \left\{I,BR,SP\right\}}Sl$$

**Figure 8:**
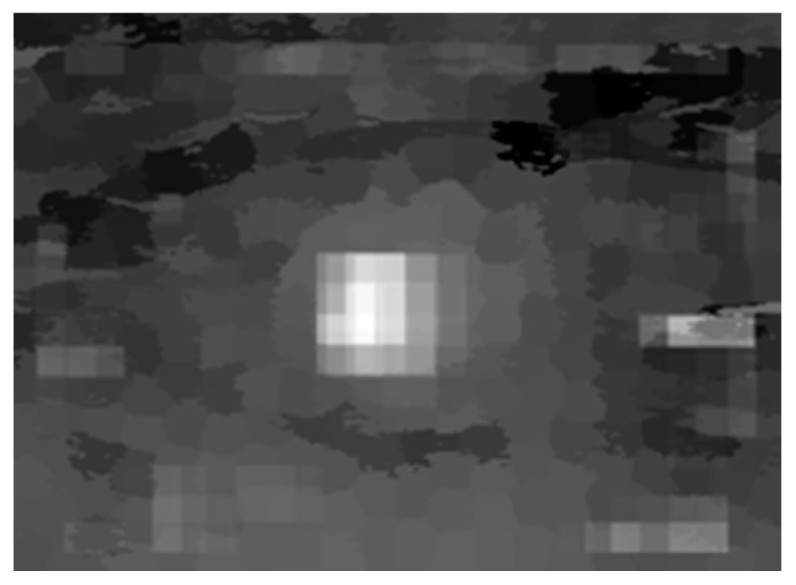
Combined saliency map.

We found above that the saliency of tumor area is greatly affected by high-intensity background areas and low-intensity background areas, respectively, in the intensity saliency map and black ratio saliency map. In addition, as illustrated in Section 2.3.2, spindly high-intensity areas are far less salient due to their smaller sizes in the superpixel saliency map. Therefore, by combining these three saliency maps, we can effectively reduce the salient values of dark background areas and high-intensity background areas, as shown in combined saliency map in Figure 8. 

### 2.5. Winner Take All

**Figure 9:**
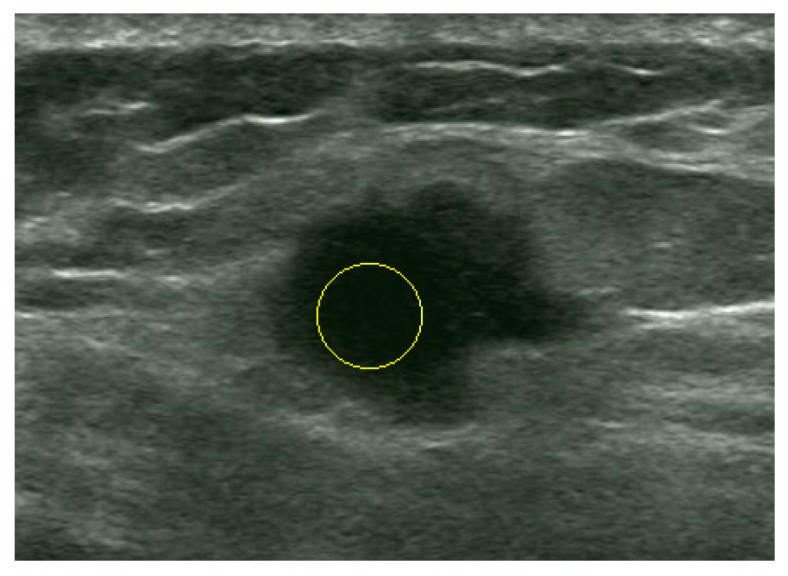
Localized tumor region.

Winner-take-all (WTA) neural networks have been extensively discussed as a way of making decisions. The maximum of the saliency map defines the most salient image localization, to which the focus of attention (FOA) should be directed (Figure 9). For our model, we used the WTA network proposed by Itti et al. [16,21], in which the saliency map is modeled as a 2D layer of leaky integrate-and-fire neurons. These model neurons consist of a capacitance that integrates the charge delivered by synaptic input, a leakage conductance, and a voltage threshold.

The saliency map neurons receive excitatory pulses from the values of saliency map xi. The potential of saliency map neurons at more salient locations hence increases faster and each neuron excites its corresponding WTA neuron independently, until the most salient saliency map neuron first reaches threshold and fires.

(10) $$\begin{array}{l} yi=\left\{ \begin{array}{ll} 0 & if\;xi<\max\left(X\right)\\ 255 & if\;xi=\max\left(X\right) \end{array}\right.\\ X=\left\{x1,x2,...,xN\right\} \end{array}$$

### 2.6. Post-Processing Operation

Based on the imaging mechanisms of the ultrasound imaging system, US breast images contain three layers, including the fat layer, mammary layer and muscle layer. The locations of suspicious lesions are in the mammary layer of US breast images, and the tumors have no junctions with the image edges. In order to overcome the interference of low-intensity areas, which are positioned in the fat layer or near the image edges, we propose an alternative strategy to automatically detect “wrong” localization by considering the position of the detected region and then selecting the “second” most probable lesion, using the information in the saliency map.

**Figure 10:**
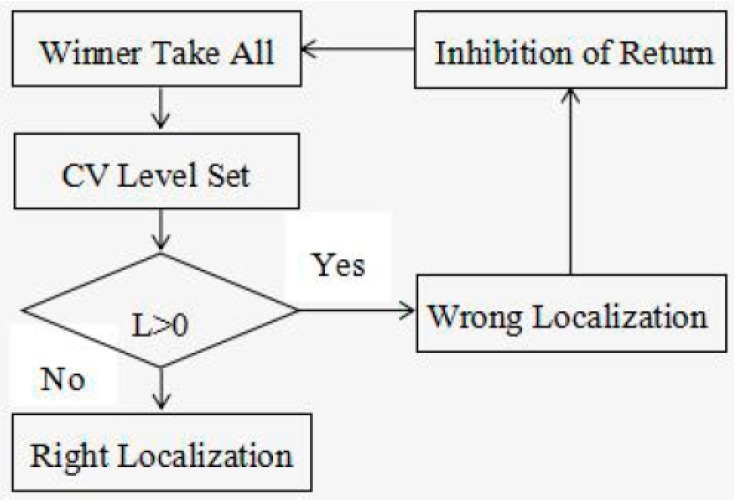
The workflow of post processing for wrong localization results.

The workflow of this strategy is depicted in Figure 10.

In order to find the second most probable lesion, the Inhibition of Return (IOR) algorithm [16] is applied in our model to inhibit the saliency of the currently attended location to 0. In Figure 10, Lj is the number of junction pixels of the segmented region and image edges. When Lj>0, the saliency of current-localized region is inhibited to 0 through the IOR algorithm and then find the second-most salient region by the winner-take-all method.

## 3. Results

An automatic localization algorithm for US breast images is tested in this section. To evaluate the proposed algorithm, 400 US breast images from the Ultrasound Department of West China Hospital of Sichuan University are tested. The proposed method successfully localized the tumors in 376 images with a high accuracy of 94.00%. 

**Figure 11:**
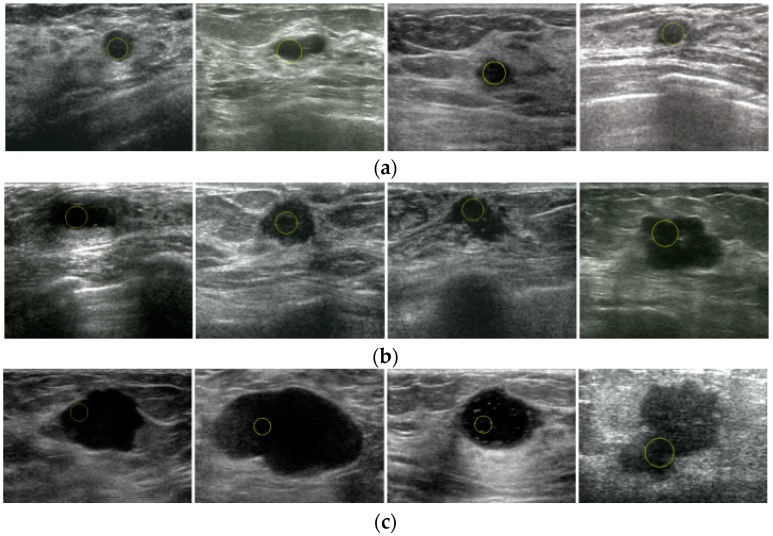
Localization results of 12 US breast images by the proposed method: (**a**) small tumors, (**b**) tumors of medium sizes, (**c**) tumors with pretty large sizes.

The results of the proposed method shown in Figure 11 demonstrate that our automatic localization scheme is valid for breast tumors with different backgrounds and sizes. The first line shows four small tumors, the second line shows four tumors of medium sizes, and the third line shows four tumors with pretty large sizes. In our method, incorporating the superpixel saliency map along with low and high-level intensity knowledge makes it possible to avoid shadowing artifacts and lowers the chance of confusing similar tumor-like structures for the lesion.

**Figure 12:**
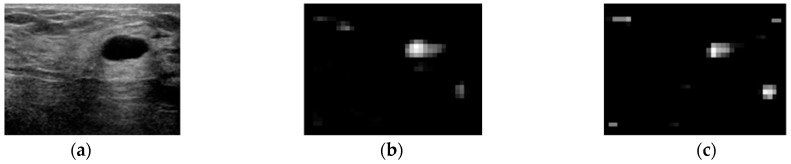

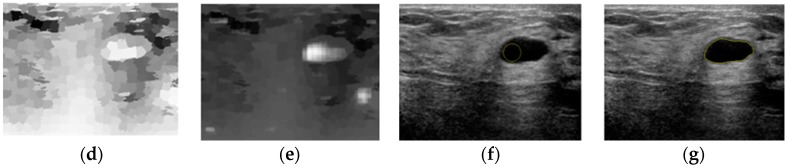
The localization and segmentation procedures of a US breast image. (**a**) Original image, (**b**) intensity saliency map, (**c**) blackness ratio saliency map, (**d**) superpixel saliency map, (**e**) combined saliency map of three features, (**f**) localization results, and (**g**) segmentation results by the CV level set.

**Figure 13:**
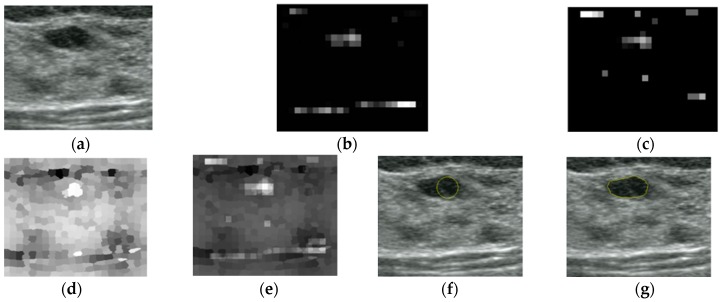
The localization and segmentation procedures of a US breast image. (**a**) Original image, (**b**) intensity saliency map, (**c**) blackness ratio saliency map, (**d**) superpixel saliency map, (**e**) combined saliency map of three features, (**f**) localization results, and (**g**) segmentation results by the CV level set.

After tumor localization by the proposed method, images are further processed through the CV level set to extract a precise contour of tumors [24]. Figure 12 and Figure 13 show the localization and segmentation procedures of two US breast images.

We can see from Figure 12b–d that the tumor has the highest saliency than other regions in these three saliency maps. It is also clear that the dark region located in the bottom-right background has equally high saliency with the tumor in the blackness ratio and superpixel saliency maps. However in the intensity saliency map, the saliency of this region is much lower than tumor, making it less salient in the combined saliency map (Figure 12e).

However, in some US breast images, the local contrast of the tumor region is lower than that of some background areas (Figure 13a). In these images, the intensity and blackness ratio features have less contributions to the localization of tumors (Figure 13b,c), in which the high-intensity bottom-right background area and the low-echo background area on the top left hold the highest saliency compared to other areas respectively in the intensity and blackness ratio saliency maps. The superpixel saliency map, in which the tumor has the highest saliency as shown in Figure 13e, devotes greatly to the localization of tumors.

**Figure 14:**
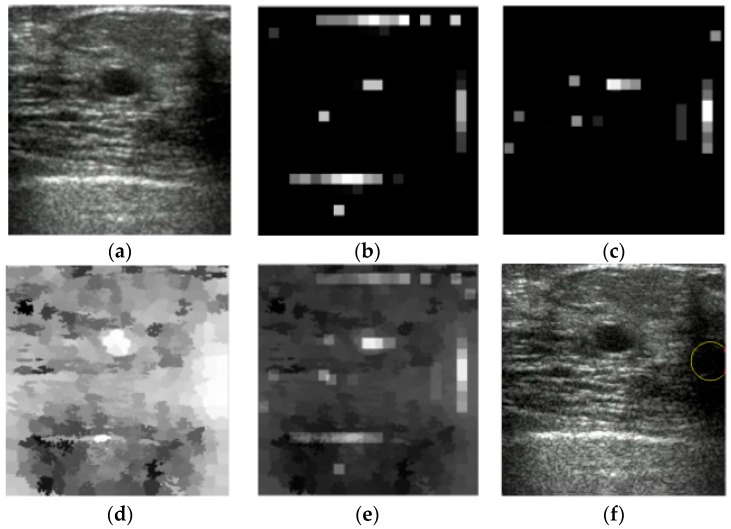
An example of wrong localization by the proposed method. (**a**) Original image, (**b**) intensity saliency map, (**c**) blackness ratio saliency map, (**d**) superpixel saliency map, (**e**) combined saliency map of three features, and (**f**) localization results.

**Figure 15:**
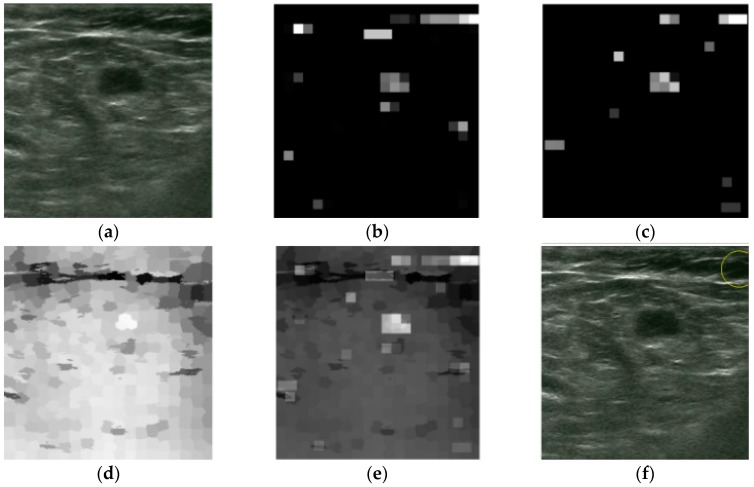
An example of wrong localization by the proposed method. (**a**) Original image, (**b**) intensity saliency map, (**c**) blackness ratio saliency map, (**d**) superpixel saliency map, (**e**) combined saliency map of three features, and (**f**) localization results.

Among the 400 tested images, we found that the tumors in 32 images have lower saliency compared with some background areas. Furthermore, in eight images, the positions of the first localized areas are in the fat layer or near the image edges. As demonstrated in Figure 14b–d, the localized dark region in these three saliency maps has equal or even higher saliency compared to tumor, making the Winner Take All network ineffective to localize the tumor. This is consistent with that of the example in Figure 15.

**Figure 16:**
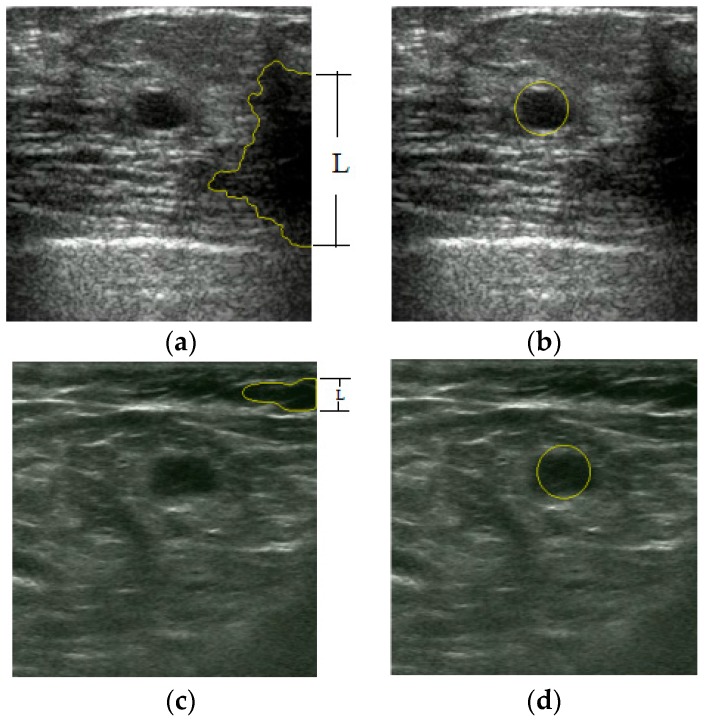
The localization procedures by post-processing operation. (**a**) Segmentation results from the CV level set in US Image 1; (**b**) right localization results by post-processing operation in US Image 1; (**c**) segmentation results from the CV level set in US Image 2; (**d**) the right localization results by post-processing operation in US Image 2.

We analyzed the localization procedures and then improved the results by implementing a post-processing operation as described in Section 2.6. The two images in Figure 15 are the segmented results from the CV level set, in which the number of junction pixels Lj>0. Therefore using the processing operation, the proposed method successfully localizes tumors in the first-wrong localization, using the information in the saliency maps, shown in Figure 16.

## 4. Discussion

In order to validate the effectiveness of the proposed scheme, it is compared with Liu’s detection method [15]. We followed the algorithm in [15] exactly and tested it using our data sets. Liu’s method was successfully executed only for 325 images Compared with Liu’s method, our method has better performance with a much higher accuracy of 94.00% compared to 81.25% (Table 1).

**Figure 17:**
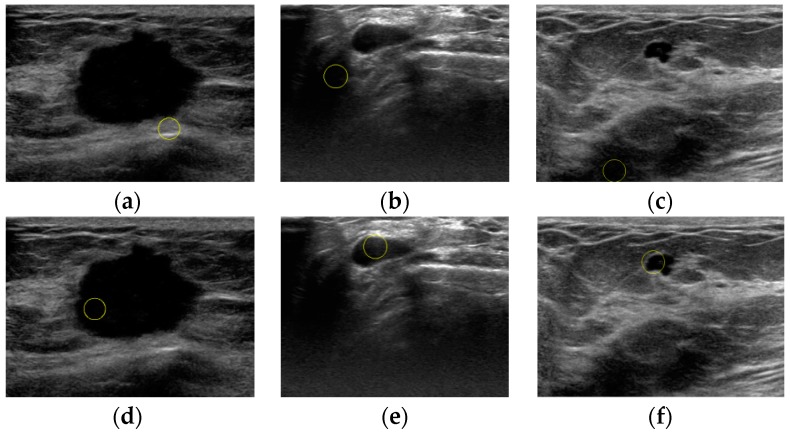
Localization results of three images in which Liu’s method failed while the proposed method succeeded. (**a**) Localization results of Liu’s method in an image with a large-sized tumor, (**b**) localization results of Liu’s method in an image with serious artifacts, (**c**) localization results of Liu’s method in an image with small tumor, (**d**) localization results of the proposed method in an image with a large-sized tumor, (**e**) localization results of the proposed method in an image with serious artifacts, and (**f**) localization results of the proposed method in an image with a small tumor.

From Table 1, the 51 tumors that are wrongly localized by Liu’s method are successfully localized by our method. Figure 17a–f show the localization results of three representative examples in this case. Liu’s method cannot separate tumor areas from normal tissues. It is unable to detect tumors with particularly large sizes, because unusually large areas are easily mistaken as background areas as a result of its classification process (Figure 17a). In addition, it is also difficult to use Liu’s method to localize tumors with serious artifacts (Figure 17b) or small sizes (Figure 17c). Liu et al. only considers local features for training SVM classifier, which consequently causes artifacts with similar textures to be more likely regarded as objects when compared with tumors with smaller sizes. In the proposed method, the saliency of tumors depends on both local and global contrast, so sizes of the tumors and serious artifacts have little effect on our localization. 

**Figure 18:**
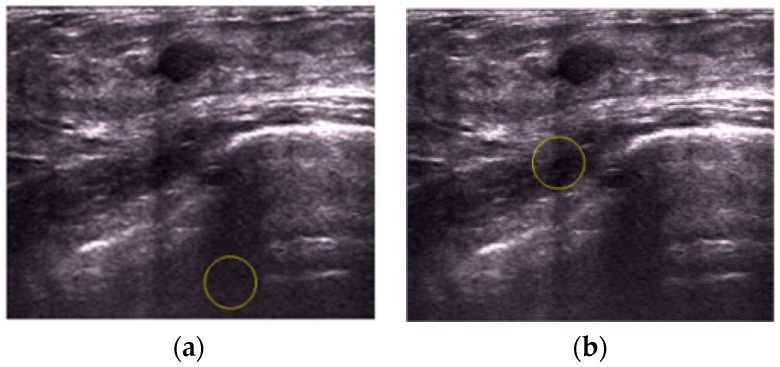
Wrong localization results of Liu’s method and the proposed method. (**a**) Localization results of Liu’s method and (**b**) the results of the proposed method.

**Figure 19:**
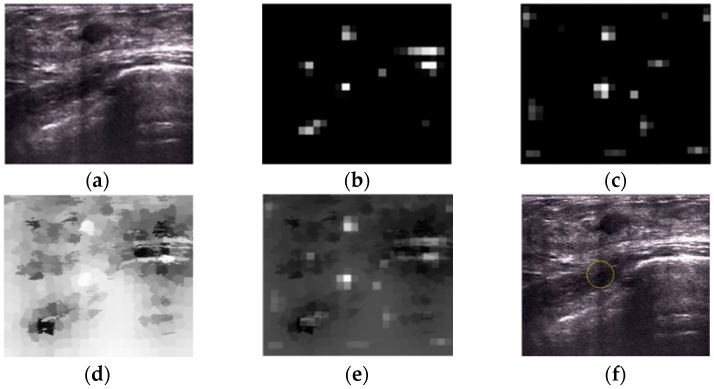
An example of wrong localization by the proposed method. (**a**) Original image, (**b**) intensity saliency map, (**c**) blackness ratio saliency map, (**d**) superpixel saliency map, (**e**) combined saliency map of three features, and (**f**) localization results.

However, two methods both fail for localization in 24 images, in which the background areas are unusually complex, like the example shown in Figure 18. The localization procedures of this image are shown in Figure 19.

We can see from Figure 19b, c that the localized region by the proposed method has a higher saliency than the tumor both in intensity and blackness ratio saliency maps. Furthermore, in a superpixel saliency map, these two regions have nearly equal saliency. Therefore, the localized region is the most salient area in this US breast image. The post-processing operation is ineffective in this case because the wrongly localized region is positioned near the center of the images and has no junction with the image edges. Thus, the post-processing operation is unable to define this localized region as “wrong localization.”

## 5. Conclusions

In this paper, a novel auto-localization method for US breast images is proposed. In the proposed method, based on the distinct characteristics of breast tumors in US images, the blackness ratio, intensity, and superpixel features are combined to compute the saliency map. The results demonstrate that the proposed method can automatically detect the tumor regions in US images with good performance and high accuracy. Comparing to Liu’s method, the proposed method performs better in US breast images with serious speckle noise or artifacts and images with particularly large-sized tumors. Combined with this method, the CV level set will no longer need the human-computer interaction to finish the extraction of tumors, which achieves the fully automatic segmentation of US breast tumors. 

However, this localization method also has some weaknesses, as it is unable to localize tumors in US breast images with complex background areas. In future work, more improvement will be employed to reduce the interference of backgrounds on the localization of tumors. Furthermore, the proposed method will be extended to other human tissues, such as the thyroid, kidney, and gallbladder.

## Figures and Tables

**Table 1:** The localization accuracy of Liu’s method and the proposed method.

Proposed Method	Liu’s Method		
	Right Localization	Wrong Localization	Sum
**Right Localization**	325	51	376
**Wrong Localization**	0	24	24
**Sum**	325	75	400
